# The Impact of Implementing the Egypt Pandemic Preparedness Plan for Acute Respiratory Infections in Combating the Early Stage of the COVID-19 Pandemic, February-July 2020: Viewpoint

**DOI:** 10.2196/27412

**Published:** 2021-05-07

**Authors:** Hanaa Abu El Sood, Shimaa Ali Abu Kamer, Reham Kamel, Hesham Magdy, Fatma S Osman, Manal Fahim, Amira Mohsen, Mohamad AbdelFatah, Mohamed Hassany, Salma Afifi, Alaa Eid

**Affiliations:** 1 Department of Epidemiology and Surveillance Preventive Sector Ministry of Health and Population Cairo Egypt; 2 Egypt Country Office World Health Organization Cairo Egypt; 3 Central Administration of Preventive Affairs Ministry of Health and Population Cairo Egypt; 4 National Hepatology and Tropical Medicine Research Institute Ministry of Health and Population Vairo Egypt; 5 Preventive Sector Ministry of Health and Population Cairo Egypt

**Keywords:** pandemic preparedness, Egypt, ARI, epidemic mitigation, COVID-19

## Abstract

This article briefly describes Egypt’s acute respiratory infection (ARI) epidemic preparedness and containment plan and illustrates the impact of implementation of the plan on combating the early stage of the COVID-19 epidemic in Egypt. Pillars of the plan include crisis management, enhancing surveillance systems and contact tracing, case and hospital management, raising community awareness, and quarantine and entry points. To identify the impact of the implementation of the plan on epidemic mitigation, a literature review was performed of studies published from Egypt in the early stage of the pandemic. In addition, data for patients with COVID-19 from February to July 2020 were obtained from the National Egyptian Surveillance system and studied to describe the situation in the early stage of the epidemic in Egypt. The lessons learned indicated that the single most important key to success in early-stage epidemic containment is the commitment of all partners to a predeveloped and agreed-upon preparedness plan. This information could be useful for other countries in the region and worldwide in mitigating future anticipated ARI epidemics and pandemics. Postepidemic evaluation is needed to better assess Egypt’s national response to the COVID-19 epidemic.

## Introduction

### Country Profile

Egypt is a transcontinental country in the Middle East. Most of the country is situated in northeastern Africa, with the Sinai Peninsula located in Western Asia. The country covers an area of 1 million km² and has coastline on the Mediterranean Sea in the north and the Red Sea in the east. Most of its population is concentrated along the banks of the Nile River and on the river's delta, and only approximately 3% of the territory is inhabited. The population of Egypt is greater than 100 million inhabitants, and the gross domestic product per capita in 2017 was US $10,799. Life expectancy was 74.4 years in females and 68.0 in males, and the under-5 mortality rate was 19.2. The main causes of death in Egypt are ischemic heart disease, stroke, and cirrhosis; the health care access and quality index was 58.0 and the governmental health spending per person was US $39 in 2018 [1].

### The Beginning of the COVID-19 Epidemic in Egypt

On December 1, 2019, a cluster of pneumonia cases of unknown cause was noticed in Wuhan, China. On December 2019, China announced an epidemic of acute respiratory disease of unknown cause [2]. As soon as the COVID-19 epidemic was announced and before the World Health Organization (WHO) announced it to be a pandemic, the Egyptian Ministry of Health and Population (MoHP) started to adapt its acute respiratory infection (ARI) pandemic preparedness plan to apply it to the anticipated pandemic.

Egypt is considered to be one of the first countries to monitor infectious diseases through a national surveillance system for reporting infectious diseases; this system has been in operation for about a century. Egypt’s national surveillance system was assessed, enhanced, and expanded to include all governmental health care facilities in 1999. The National Egyptian Electronic Disease Surveillance System (NEDSS) targets 40 communicable diseases, including ARIs, and it includes an electronic reporting element [3]. A comprehensive network of epidemiological and laboratory vertical systems for reporting ARIs was developed in 2009, along with an alert system for early detection of novel respiratory viruses. The network covers the entire country and comprises surveillance systems targeting severe acute respiratory infections (SARIs), influenza-like illness (ILI), pneumonia, avian influenza, and Middle East respiratory syndrome coronavirus (MERS-CoV). Event-based surveillance was introduced in Egypt in 2009 in response to the 2009 H1N1 pandemic to aid timely detection of and response to possible epidemics.

The aims of this viewpoint are to review and discuss the preventive and control measures that have been implemented by the Egyptian MoHP in response to the COVID-19 pandemic and to share Egypt’s experience with public health practitioners and authorities to enable better response to such events in the future.

The specific objective of the preparedness plan for response to COVID-19 was to reduce morbidity and mortality in the event of a COVID-19 epidemic in Egypt.

## Methodology

In this report, we use two approaches: to discuss the interventions implemented in the COVID-19 epidemic in Egypt as a part of the preparedness plan and to describe the epidemic situation in its early stage in an attempt to link it to the interventions performed.

To describe the ARI epidemic preparedness and containment plan in Egypt, all documents addressing the ARI epidemic preparedness and response plan and their updates were reviewed, including the ARI case management plan, ARI and Influenza Pandemic Preparedness Plan, and national disease surveillance guidelines. In addition, the key studies and publications describing the situation of the early stage of the COVID-19 epidemic were reviewed to describe the epidemic situation.

National surveillance data on all patients in Egypt with COVID-19 from February to July 2020 were obtained from the national disease surveillance database. Web-based data sources were consulted regularly to collect data published on the early stage of the epidemic in Egypt. Descriptive data analysis was performed to assess the response to the epidemic in Egypt.

## Egypt’s ARI Pandemic Preparedness and Containment Plan

Egypt’s ARI preparedness plan was first developed in 2007 in collaboration with WHO Egypt. It was activated in 2009 during the H1N1 pandemic, updated in 2019, and then adapted to the COVID-19 pandemic [4].

The pandemic preparedness plan was activated early, before the introduction of SARS-CoV-2 into the country, when the first positive case was identified among contacts of a Chinese woman who tested positive after returning to China from a short business trip to Cairo.

The plan includes five pillars:

1. Crisis management
2. Enhancing surveillance systems and contact tracing
3. Case and hospital management
4. Raising community awareness
5. Quarantine and entry points

### Crisis Management

A pre-established Supreme National Committee for crisis management was activated early in the pandemic. Members of the committee are Ministers of relevant Ministries, including the Ministry of Health, Ministry of Agriculture, Ministry of Local Development, Ministry of Environment, Ministry of Defense, Ministry of Foreign Affairs, Ministry of General Information Authority, General Administration of Veterinary Affairs, Crisis Management Room at Council of Ministers, and Ministry of the Interior, Preventive and Endemic Affairs of Ministry of Health. This committee was and is still responsible for deciding on the necessary preventive and control measures based on the rapid changes in the pandemic situation globally and in Egypt.

The crisis management committee of the MoHP was assembled before the introduction of the virus to Egypt. The committee included all sectors concerned within the ministry (preventive sector, curative sector, health care and nursing sector, general authority for health insurance, general authority for hospitals and educational institutes, central administration of medical medicine, central administration of preventive affairs, General Secretariat of Specialized Medical Centers, Central Administration of Pharmacy, General Directorate of Hospitals, General Department of Chest Diseases, General Department of Infectious Diseases, General Administration of Veterinary Affairs Mechanism, Epidemiology and Surveillance, the official spokesperson of the Ministry of Health, and a representative of the Supreme Council of Universities). The committee is responsible for monitoring the epidemiologic situation on a 24-hour basis, implementing all preventive and curative measures to contain the disease, ensuring the preparedness and response of emergency teams 24 hours per day, establishing follow-up treatment protocols, and ensuring the availability of medical supplies necessary for prevention and case management and equipment and supplies for intensive care units.

### Enhancing ARI Surveillance Systems

#### Case Definitions

NEDSS, ILI, SARI, and pneumonia and mortality surveillance systems were activated through 2 days of training for surveillance teams in all governmental health care facilities conducted at the central and regional levels. Guidelines for COVID-19 epidemic response were developed and distributed at all health system levels, and teams were instructed to report ARIs on a daily basis. Case definitions were developed using the WHO COVID-19 case definition [5] and distributed to all governmental and private health care facilities for reporting any suspected cases. The case definition was updated 3 times to increase the sensitivity for case detection as the epidemic progressed.

The updated COVID-19 case definitions as of June 2020 are outlined below.

A *suspected case* is defined as anyone who suffers from acute respiratory symptoms (cough, shortness of breath) or fever ≥38 °C or both with no other reasons, or anyone with any of the following conditions within 14 days before symptoms:

1. History of travel to a country or region that proves wide community spread or limited local transmission of COVID-19
2. Contact with a confirmed case with COVID-19
3. Contact with a person with acute respiratory symptoms (cough, shortness of breath) or a fever ≥38 °C and who is epidemiologically related to a place or region (locally or internationally) with epidemic outbreaks of COVID-19 but not yet confirmed by a laboratory
4. Health care workers or workers in a health care facility with reported confirmed cases of COVID-19 or a patient with SARI with fever ≥38 °C with one of the symptoms of acute respiratory disease (cough, shortness of breath) and the cause of the pathological condition could not be identified

If suspicion of the above is not verified, consider any person with at least two of the following clinical characteristics:

1. Fever, severe respiratory symptoms, or both
2. Computed tomography scan for chest (if not available; normal x-rays are performed on the chest) with diagnostic properties of COVID-19
3. Normal or low leukocyte count with lymphocytopenia

A *confirmed case* is defined as a person with laboratory confirmation of COVID-19 infection by real-time polymerase chain reaction (RT-PCR).

COVID-19 was added to the list of NEDSS-reportable diseases, and a data collection was developed and added to the web-based data screens. Suspected and confirmed cases of COVID-19 are entered at all governmental hospitals to provide a regular description of the epidemic situation in Egypt, for contact tracing, and for future predictions. Daily reports are developed and shared with relevant stakeholders.

The International Health Regulations unit at MoHP is in direct contact with WHO Egypt and Eastern Mediterranean Regional (EMR) Office for daily reporting, regular sharing of global- and country-level information, updated recommendations, and viral genetic mutations.

The event-based surveillance was expanded to include the entire country, and teams at all levels are immediately reporting alert signals to the central level. Signal verification and case detection are performed for all received alert signals.

#### The Role of Laboratories in Epidemic Mitigation

Specimens are collected from all suspected cases for testing at regional laboratories in governorates and central laboratories in Cairo. Testing results are monitored daily and distributed to affiliated governorates and hospitals. Genetic mutation is monitored regularly at the global and regional levels.

COVID-19 and influenza testing kits and reagents are secured at the Central Public Health Laboratory and regional laboratories. Training was provided to laboratory specialists and technicians at the governorate level in specimen collection, archiving, and transfer. Specimens are shared regularly with WHO reference laboratories.

#### Contact Tracing

Evidence from the COVID-19 response in China has indicated that efficient contact tracing can enable early detection and isolation of cases and can substantially reduce disease transmission [6]. Because contact tracing is a crucial part of COVID-19 epidemic control in conjunction with case finding, Egypt was able to apply this strategy early in the pandemic, when the number of cases was small. In Egypt, contacts were classified as close and casual contacts according to the European Centers for Disease Control and Prevention definition and traced to provide contacts with information on self-quarantine, proper hand hygiene, and respiratory etiquette measures and to advise them on what to do if they developed symptoms. Contacts with symptoms undergo laboratory testing by PCR in a timely fashion for early case detection [7]. Responsibilities for contact tracing and management were developed and distributed at all levels of the health system.

#### Case and Hospital Management

Intensive care units (adult and pediatric) and hospitals with ventilator capacity (adult and pediatric) were identified for the management of COVID-19 cases. A referral system between hospitals was defined, and information was distributed to all governorates and hospitals. Training was provided for all hospital physicians in case detection using the case definitions, triage, and protocol for management of acute respiratory infections. Continuous supervision and monitoring from the central level of assigned hospitals was performed to evaluate hospital performance in dealing with cases of acute respiratory symptoms. Chest consultants were assigned for management of severe cases. A manual was developed for treatment of patients at different disease stages and severity. This manual is being revised and updated regularly as new information becomes available.

### Raising Community Awareness

A hotline was activated and WhatsApp was used 7 days per week to answer questions, respond to requests and complaints and coordinate patient transfer and hospital admissions. In addition, Facebook, radio, and television broadcasts were used to raise community awareness of the current status of the COVID-19 pandemic in Egypt, disease prevention, and home management of patients. Printed posters were developed and distributed at the intermediate and peripheral health system and hospital levels. The printed material included 3000 posters for case definition, 30,000 brochures of fact sheets, 2000 case management booklets, and 30,000 brochures on how to manage patients at home, in addition to >2 million posters on preventive measures at home, in the community, and at health care facilities. The printed materials were delivered to all governmental and private health facilities and communities.

### Quarantine and Points of Entry

Brochures were distributed to staff at all points of entry, including the definitions of suspected and confirmed cases of COVID-19. Travelers arriving to Egypt were assessed, and suspected cases were transferred to isolation hospitals as preventive measures. During the period from January to July 2020, 91,787 travelers arrived via different points of entry. Of these 91,787 travelers, 1616 (1.8%) had elevated temperature or respiratory symptoms detected at the checkpoints and were transferred to hospitals for evaluation and treatment. All asymptomatic arrivals (90,171) were tested by rapid testing. Of them, 2329/90,171 (2.6%) tested positive and were sent for PCR confirmation testing. Of the 2329 persons who tested positive by rapid testing, 966 (41.5%) tested positive by PCR and were quarantined to prevent spread of the disease and for follow-up. All remining travelers (87,842) were sent home for home isolation and were followed up on a daily basis by telephone to determine any symptoms appeared.

## Source of Data

We used data from the NEDSS from February to July 2020. Descriptive data analysis was performed by time, person, and place using Epi Info, version 7 (US Centers for Disease Control and Prevention). Attack rate was calculated as the total number of confirmed cases/100,000 population with the population data for 2020 obtained from the Department of Information Center at MoHP.

## Situation During the Early Phase of the Epidemic

**Figure 1:**
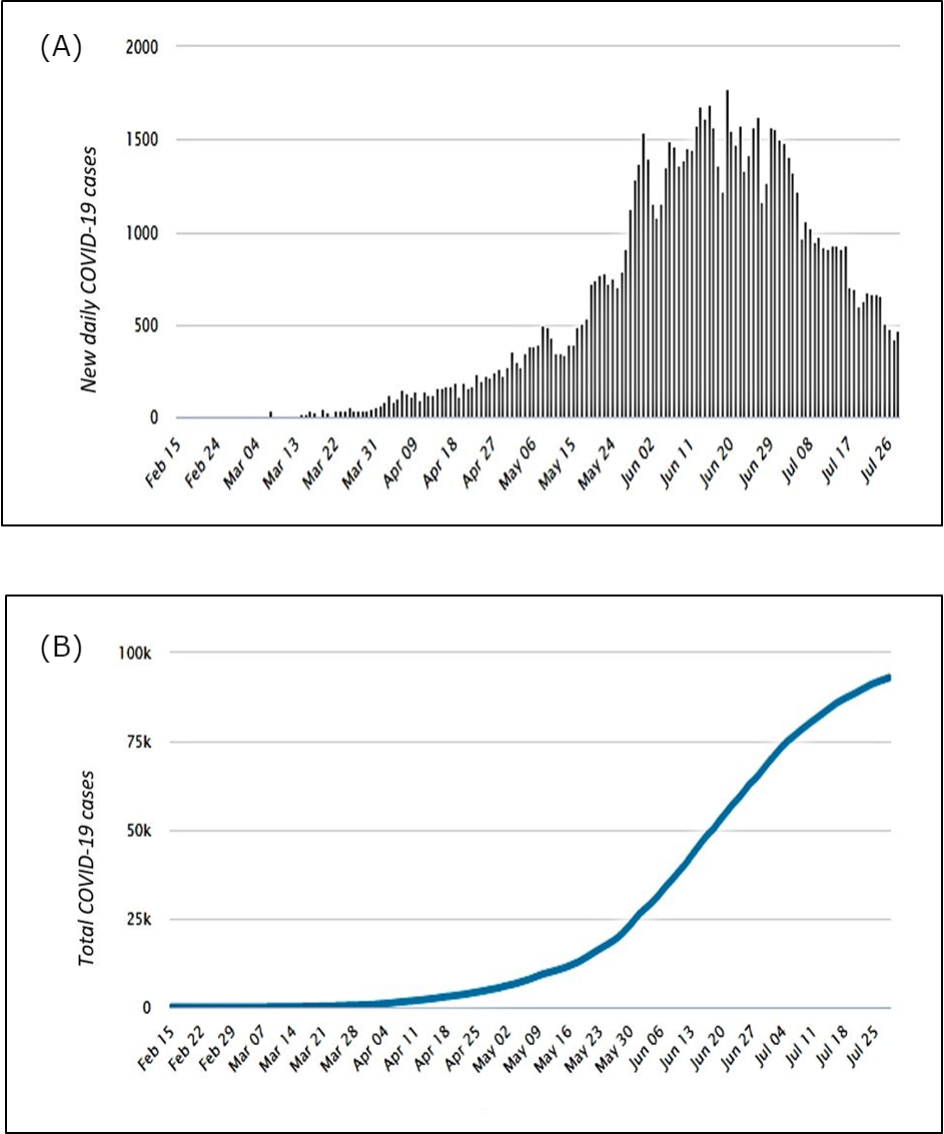
(A) Daily new and (B) total numbers of COVID-19 cases in Egypt from February to July 2020 [9].

**Figure 2:**
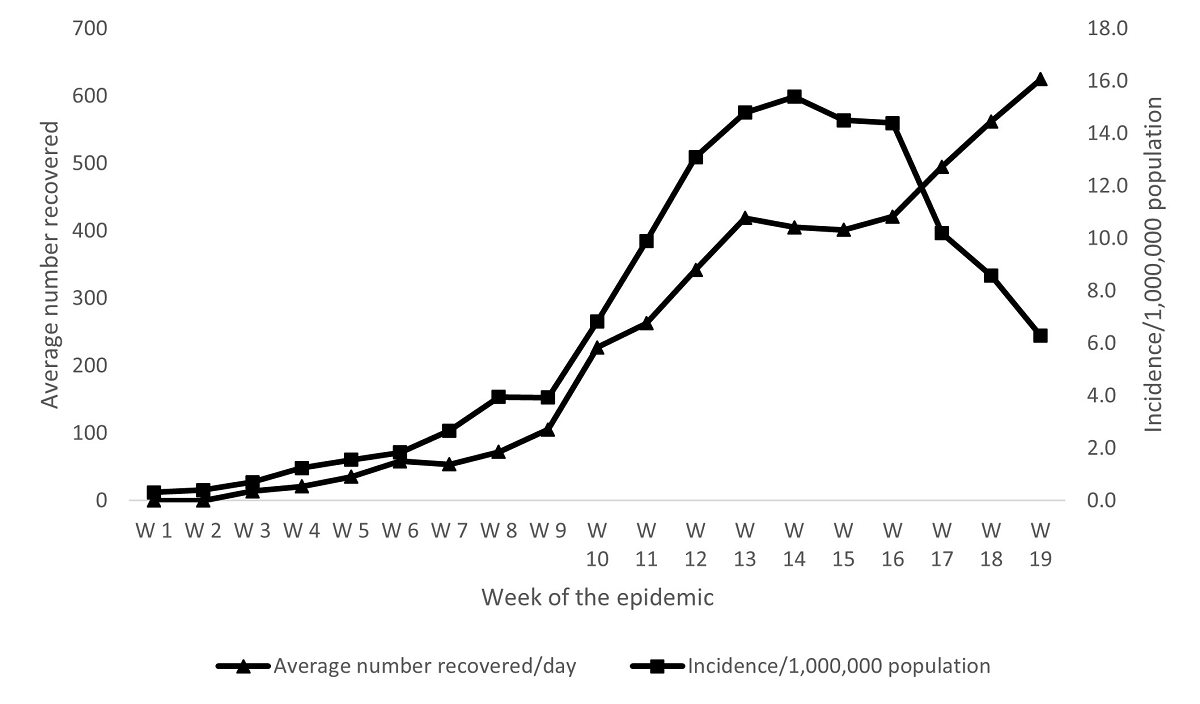
Incidence rate of COVID-19 and average number of recoveries in Egypt, February to July 2020. Source: Ministry of Health and Population daily COVID-19 report.

According to MoHP senior officials, Egypt passed the first wave of the epidemic peak and entered the flattening phase of daily cases in mid-June 2020, with the highest number of cases in one day (1774) reported on June 20. The number of cases started to gradually drop during July, August, and possibly through September, with the lowest number of cases (112) reported on August 5 (Figures 1 and 2). Modeling studies suggested that Egypt had succeeded in delaying the peak of the COVID-19 curve after the seventh week, with no exponential growth of transmission rate identified [8].

The overall number of cases by week started to rise in early May and peaked in mid-June, after which it started to decline but did not reach the baseline (Figure 1, A and B) [9]. The mean weekly growth rate from February to May 2020 was 0.35 (SD 0.33), which declined over time from 1.18 in early March to 0.13 at the end of May. Similarly, the reproductive number (R_0_) and herd immunity threshold declined from 6.5 to 1.6 and from 85% to 39%, respectively; meanwhile, the doubling time increased from 1.8 days to 15.6 days (Table 1) [10].

**Table 1:** Reproductive number (R_0_) and corresponding herd immunity threshold of COVID-19 in Egypt, February-May 2020 [10].

Date (2020)	Growth rate (weekly)^a^	Doubling time (days)^b^	Reproductive number^c^	Herd immunity threshold (%)^d^
February 28	0	0	1	0
March 7	1.18	1.8	6.5	85
March 14	0.79	2.7	4.7	79
March 21	0.44	4.8	3.1	67
March 28	0.29	7.4	2.3	57
April 7	0.34	6.3	2.6	61
April 14	0.31	6.8	2.5	59
April 21	0.18	11.6	1.9	46
April 28	0.16	13.4	1.7	42
May 7	0.2	10.5	1.9	49
May 14	0.14	15.2	1.6	39
May 21	0.13	15.6	1.6	39

^a^Mean 0.35 (SD 0.33).

^b^Mean 8.75 (SD 4.85).

^c^Mean 2.6 (SD 1.55).

^d^Mean 52 (SD 22).

**Figure 3:**
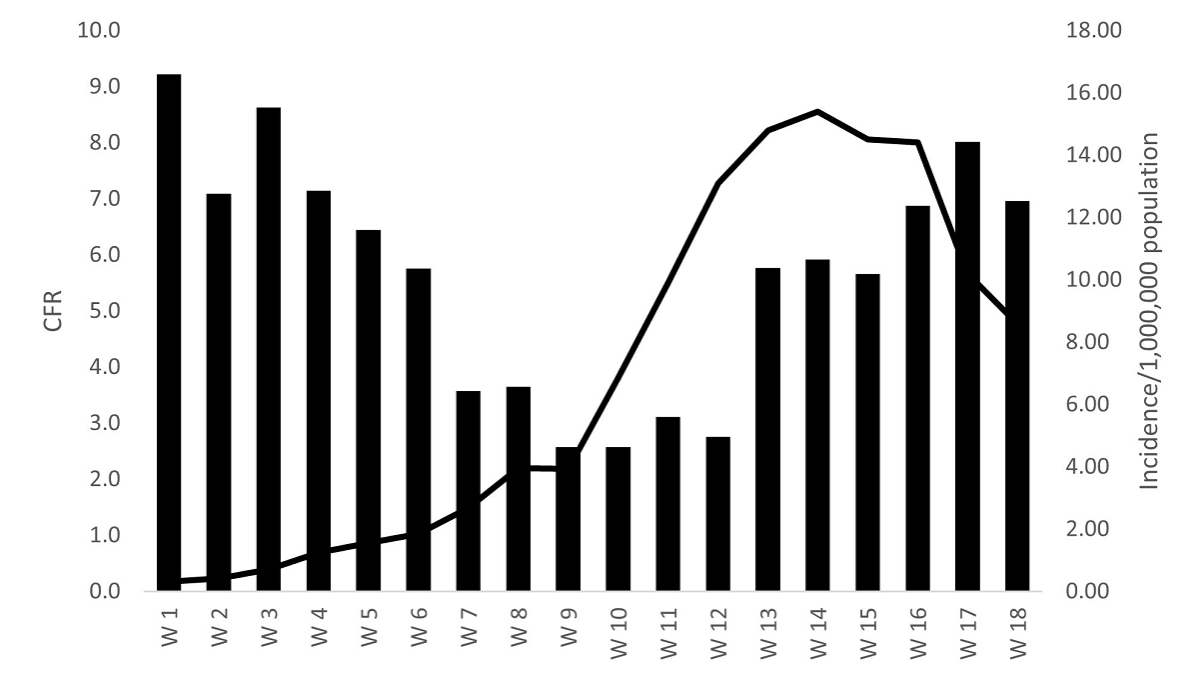
COVID-19 incidence rate and case fatality rate in Egypt, February to July 2020. Source: Ministry of Health and Population daily COVID-19 report. CFR: case fatality rate.

Incidence of COVID-19 in Egypt by week peaked in the 14th week after the first case was confirmed in Egypt (15.4/1,000,000 population) and declined to 6.29 in the 19th week after the first case; meanwhile, the average number recovered per week constantly increased, reaching 625.0 by the 19th week after the first case was reported (Figure 2). The case fatality rate showed a pattern of 2 peaks, 1 each in weeks 1-4 and weeks 16-18, with an increase after the decline in weeks 9-12, ranging from 2.57% in weeks 9-10 to 9.22% in week 1 (Figure 3).

Overall, 102,789 COVID-19 cases were reported to NEDSS in the study period, including 78,048 (43.2%) confirmed COVID-19 cases, giving an incidence of 77.0 cases/1,000,000 population, and 3457 (4.4%) deaths were reported due to COVID-19. Of the 78,048 case patients, 44,969 (57.6%) had mild symptoms and were identified in outpatient clinics. The middle age group (35-60 years) was the most affected; meanwhile, the disease was rare in people <15 years of age, representing 2.6% of all cases (2043/78,048), with slightly more male patients (40,621/78,048, 52%) than female (37,422/78,048, 48%). Of the 78,048 confirmed cases, 39,964 (51.2%) were admitted to hospital for health care, while 33,037 (48.8%) were isolated at home or youth hostels for follow-up (Table 2).

Among the 54,300 cases for whom clinical data were available, the most prominent symptom was fever (45,188, 83.2%), followed by cough (39,549, 72.8%), dyspnea (27,009, 49.7%), and sore throat (26,330, 48.5%). Of the 12,661 confirmed cases for whom comorbidity data were available, 6845 (54.1%) had a history of diabetes and 5816 (45.9%) had cardiovascular disease, while only 274 (2.2%) were obese and 279 (2.2%) were pregnant (Table 2).

**Table 2:** Demographic, epidemiologic, and clinical characteristics of patients with confirmed COVID-19, National Egyptian Disease Surveillance, February to July 2020.

Characteristic			Value, n (%)
Suspected cases			102,789 (N/A)^a^
Confirmed cases			78,048 (43.2)
**Source**			
	Inpatient	44,969 (57.6)	
	Outpatient	33,079 (42.4)	
**Age group (years)**			
	<1	223 (0.3)	
	1-4	552 (0.7)	
	5-14	1268 (1.6)	
	15-34	20,197 (25.9)	
	35-64	44,948 (57.6)	
	≥65	10,855 (13.9)	
**Gender**			
	Male	40,621 (52)	
	Female	37,422 (48)	
**Clinical picture^b^**			
	Fever	45,188 (83.2)	
	Cough	39,549 (72.8)	
	Difficulty breathing	27,009 (49.7)	
	Sore throat	26,330 (48.5)	
	Joint pain	18,287 (33.7)	
	Diarrhea	6260 (11.5)	
	Vomiting	4730 (8.7)	
	Pneumonia	23,503 (43.3)	
**Comorbidity^c^**			
	Diabetes	6845 (54.1)	
	Cardiovascular disease	5816 (45.9)	
	Chronic obstructive pulmonary disease	2620 (20.7)	
	Renal	630 (5.0)	
	Liver	491 (3.9)	
	Pregnant	279 (2.2)	
	Obese	274 (2.2)	
	Immunocompromised	226 (1.8)	
Isolation			
	Hospital isolation	39,964 (51.2)	
	Youth hostel	8380 (10.7)	
	Home isolation	29,704 (38.1)	
	Case fatality	3333 (4.3)	

^a^N/A: not applicable.

^b^Clinical data available for 54,300 cases.

^c^Comorbidity data available for 12,661 cases.

**Figure 4:**
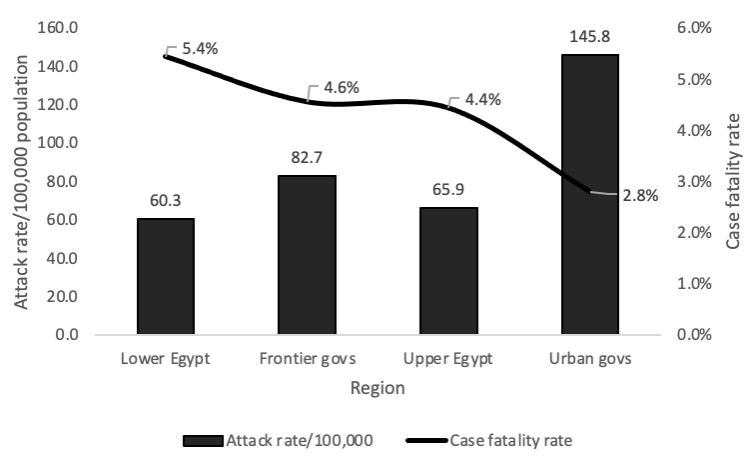
Distribution of COVID-19 attack rates and case fatality rates by region, National Egyptian Disease Surveillance, February to July 2020. Govs: governorates.

Incidence rate differed by region of residence, ranging from 60.3 in Lower Egypt to 145.8/1,000,000 population in the urban governorates, while the case fatality rate (CFR) ranged from 2.8% in urban governorates to 5.4% in Lower Egypt governorates. Data analysis indicated that highest incidence rate and lowest CFR were identified in the urban governorates (Figure 4).

## Conclusions

Egypt succeeded in containing the early stage of the COVID-19 epidemic. Containment measures taken in the early stage of the epidemic depended mainly on early case detection and tracing and isolation of infected persons as well as on raising community awareness to stop the disease from spreading. Strengthening of mitigation efforts began when community-wide transmission occurred in the form of proper case management and coordination of different interventions to slow the spread of COVID-19 and mitigate its effects on the health care system and community. The ARI pandemic preparedness plan proved effective in epidemic mitigation, with high commitment from all partners. Postepidemic performance evaluation is needed to better assess Egypt’s national response to the COVID-19 epidemic.

## Abbreviations

ARI: acute respiratory infection
CFR: case fatality rate
ILI: influenza-like illness
MERS-CoV: Middle East respiratory syndrome coronavirus
MoHP: Ministry of Health and Population
NEDSS: National Egyptian Electronic Disease Surveillance System
RT-PCR: real-time polymerase chain reaction
SARI: severe acute respiratory illness
WHO: World Health Organization
